# Acute Renal Failure due to a Tobramycin and Vancomycin Spacer in Revision Two-Staged Knee Arthroplasty

**DOI:** 10.1155/2018/6579894

**Published:** 2018-07-02

**Authors:** Ronak A. Patel, Hayden P. Baker, Sara B. Smith

**Affiliations:** College of Medicine, University of Illinois at Chicago, Chicago, IL, USA

## Abstract

Two-stage revision total knee arthroplasty (TKA) is the standard of care for prosthetic joint infections. The first stage involves removal of the infected prosthesis and placement of an antibiotic impregnated cement spacer; following a period ranging from 4 weeks to 6 months, the spacer is then removed and replaced with a permanent prosthesis. The advantage to this approach is that antibiotic impregnated spacers provide supratherapeutic levels in the joint without toxic accumulation in serum. However, it remains important for physicians and pharmacists to be aware of antibiotic associated complications in knee revisions. We present a case of a two-stage revision total knee arthroplasty in which a cement antibiotic spacer caused acute renal failure and ultimately resulted in persistent chronic kidney disease without hemodialysis at 2 months' follow-up. Our case reports the third highest serum tobramycin (13.7 mcg/ml) and second highest serum creatinine (8.62 mg/dl) for patients experiencing ARF due to an antibiotic spacer in two-stage revision TKA.

## 1. Introduction

Periprosthetic joint infections (PJI) are a common complication of arthroplasty, with an incidence of 1-3% [1, 2]. Typical treatment involves a two-staged revision total knee arthroplasty, whereby the infected prosthesis is removed and replaced with a temporary antibiotic impregnated spacer. Parenteral antibiotics are also administered in order to create supratherapeutic levels [3]. Following a period ranging from 4 weeks to 6 months, the spacer is removed and replaced with a permanent prosthesis [4–7]. Using this method, literature describes success in approximately 90% of patients, demonstrating the efficacy of the procedure [1, 5, 8]. However, complications may include acute kidney injury (AKI) and reinfection [1, 4–6].

Nephrotoxic antibiotics are mixed with bone cement in the creation of temporary spacers to treat PJI [5, 6]. As a result, multiple studies show that AKI during two-stage revision TKA is both a common and underreported complication, with an incidence of 4.8-26% [5, 6, 9]. The AKI is often mild and transient [5, 6]. However, there have been case reports and case series detailing progression of AKI to acute renal failure (ARF) requiring hemodialysis in the setting of a two-stage revision TKA [5]. Most cases resolve with kidney function returning to baseline [5]. We present a case of a tobramycin and vancomycin spacer causing ARF requiring hemodialysis with resultant Stage IV CKD at 2 months' follow-up. Our case reports the third highest serum tobramycin and second highest serum creatinine for patients experiencing ARF due to an antibiotic spacer in two-stage revision TKA.

## 2. Case Description

A 65-year-old male with a history of multiple periprosthetic infections of the left knee presented for the first stage of his revision TKA. His past medical history included diabetes, obstructive sleep apnea, congestive heart failure, and gastroesophageal reflux disease. Previous surgeries included a lumbar spinal fusion and multiple failed revision two-stage TKAs to treat his periprosthetic infection. Medications at the time included furosemide, gabapentin, carvedilol, lansoprazole, docusate, and enalapril. There was no documented history of allergies or complications with anesthesia.

On the operative day, the patient was brought to the operating room and cephalexin 2 mg was administered. Upon opening the left knee, cloudy fluid was appreciated and sent for culture and sensitivities. Both the infected cement spacers on the femur and tibia were debrided and irrigated. Tobramycin and vancomycin cement mixture formed the new spacer. A total of 5 bags of Simplex P (Stryker, Mahwah, NJ) cement were mixed with 26.4 g of tobramycin and 9 g of vancomycin. Intraoperatively, records showed brief episodes of hypotension on induction requiring 3 pressors. For the duration of the case, he required intermittent pressure support with a total of phenylephrine 360 mcg, epinephrine 30 mcg, and norepinephrine 36 mcg. He was extubated and transferred to the recovery room in stable condition. His medications postoperatively included celecoxib 200 mg BID and aspirin 325 mg BID, and he was continued on lansoprazole.

Vancomycin 2 g IV every 12 hours and piperacillin-tazobactam 3.375 g IV every 6 hours were started. Cultures revealed the joint to be infected with* Corynebacterium striatum*, and as a result IV piperacillin-tazobactam was discontinued.

**Figure 1:**
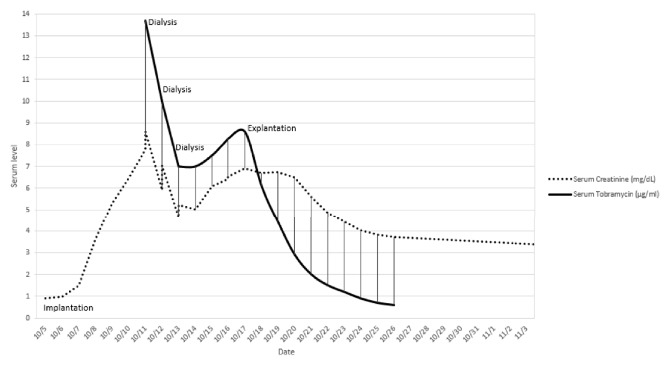
*Serum Tobramycin and Creatinine Levels*. Serum tobramycin and creatinine levels trended from surgical implantation of the antibiotic spacer on 10/5, to 3 sessions of dialysis on 10/11, 10/12, and 10/13, to explanation and replacement with a 4 g cephazolin spacer on 10/17.

On postoperative day (POD) 2, he developed a nonoliguric AKI from a baseline creatinine of 0.9 mg/dl to 1.5 mg/dl. As the AKI progressed, on subsequent days, the consulting nephrology team speculated that the etiology was multifactorial, likely secondary to ATN from intraoperative hypotension and nephrotoxic medication side effects from his celecoxib, lansoprazole, or IV vancomycin. On POD 3, a random vancomycin level was drawn at 53.8 mcg/ml with subsequent discontinuation of IV vancomycin and celecoxib. He was then started on doxycycline 100 mg BID. On POD 6, his AKI progressed to a peak creatinine of 8.62 mg/dl and hyperkalemia at 6.3 mmol/L with marked ECG changes prompting administration of calcium gluconate and kayexalate with emergent hemodialysis and ICU transfer. Tobramycin levels were drawn, and results showed 13.7 mcg/ml. Hemodialysis was repeated on POD 7 and 8 and tobramycin levels downtrended as seen in Figure 1. Following the 3 total sessions of hemodialysis, the patient was able to produce adequate volumes of urine, and hemodialysis was discontinued. Following the cessation of hemodialysis, tobramycin levels began to uptrend. At that time, the decision was made to explant the antibiotic impregnated spacer containing vancomycin and tobramycin.

The antibiotic spacer was subsequently explanted on POD 13 with a reimplantation of a spacer containing 4 g cefazolin. In the following days, he experienced a marked reduction in creatinine to 3.72 mg/dl and tobramycin level to 0.6 mcg/ml upon discharge. At two months' follow-up, his serum creatinine downtrended and stabilized to 2.28 mg/dl without evidence of hyperkalemia or oliguria.

## 3. Discussion

PJI is a common complication among patients undergoing TKA [5, 6]. Treatment includes two-stage TKA with an antibiotic spacer followed by replacement with a permanent prosthesis and is successful in approximately 90% of patients [1]. However, a two-stage revision TKA bears a significant risk of AKI with potential for progression to ARF [10, 11].

Antibiotic spacers used to treat PJI have their own host of side effects. In our case, both vancomycin and tobramycin have known nephrotoxicity, which most likely contributed to our patient's ARF (Naranjo Score=6) [12–14]. Antibiotic choice within the spacer is a crucial step to successful treatment of PJI. Aminoglycosides alone or in combination with vancomycin are the most frequently used [4, 5, 13]. Although there is little data comparing different mixtures of antibiotics, this combination provides both Gram-negative and Gram-positive coverage to treat PJI [1].

The incidence of AKI in two-stage revision TKA varies from 4.8 to 26% [5, 6, 8, 9]. Data surrounding potential risks are limited but may include increased dosage of tobramycin and vancomycin in the spacer, administration of nephrotoxic IV antibiotics, low hemoglobin, high patient BMI, nonsteroidal use, intraoperative hypotension, and concomitant CKD among other factors [5, 6, 8, 15, 16]. Multiple studies suggest that IV antibiotics and intraoperative hypotension requiring vasopressors are not significant risk factors for the development of AKI in this setting [6, 8, 15]. However, our patient experienced several of the theoretical risk factors including intraoperative hypotension requiring vasopressors, NSAID use, low hemoglobin, high patient BMI, and a high dose antibiotic spacer that may have contributed to his ARF.

The elution characteristics from the spacers depend on a variety of factors including the cement type as well as amounts and types of antibiotics [17, 18]. For there to be clinically relevant elution, greater than 3.6 g of antibiotic per 40 g of cement needs to be present [19]. Various elution curves have been theorized, with a majority suggesting peak values during POD 1-2, followed by a gradual decline to steady state [17]. Increased antibiotic content within spacers may contribute to duration of elution, but other factors are not well studied [18]. Elution duration can range from weeks to months [19].

There are no current standardized recommendations for the quantity of antibiotic in each spacer. Earlier studies by Springer et al. and Evans et al. indicated high dose antibiotics to be both safe and efficacious [9, 20]. More recent studies question the notion, stating the risk of adverse effects increases with increasing doses of antibiotic [6]. Doses of tobramycin >4.8 g increased odds of AKI in patients by 5.87 (95% CI, 1.43-24.19; P = .01) and may be dosage dependent, with every 1 g increase of antibiotic increasing the odds of AKI by 1.24 (95% CI, 1.00-1.52; P = .049) [6]. General guidelines suggest low dose spacers contain <2.0 g of antibiotic per 40 g of cement, and high dose spacers contain >3.6 g of antibiotic per 40 g of cement [14]. There is data to suggest a lower dose spacer increases the risk of reinfection, with Geller et al. suggesting the odds of failure are higher (OR, 0.82; 95% CI, 0.70- 0.96; P = .01) with low dose antibiotics at 1 year and 2 years (OR, 0.83; 95% CI, 0.71-0.96; P = .01) [8]. The dosage our patient received was within recommendations and constituted high dose antibiotics. Overall, he received 26.4 g tobramycin and 9 g vancomycin total in 5 bags of 40 g cement.

It is also unclear whether antibiotics contribute to changes of elution from cement spacers, with the most studied combination being tobramycin and vancomycin [13, 21]. In an in vitro analysis by Klekamp et al., the elution of vancomycin failed to affect the elution of tobramycin and vice versa [13]. In a similar analysis by Penner et al., the combination antibiotic increased elution of tobramycin by 68% and vancomycin by 103% compared to each antibiotic alone [18]. Penner et al. speculated that increased amounts of antibiotic within the cement created porosity and increased surface area, allowing for higher elution rates [18]. In vivo, Masri et al. suggested increasing tobramycin levels provided increased elution of vancomycin [19]. In the context of various conflicting studies, the impact of combination of tobramycin and vancomycin on each antibiotic's elution characteristics in our patient cannot be reliably determined.

Treatment for AKI remains conservative with the determination of fluid status and supportive care [5, 6]. Most patients experience AKI due to reduction in renal blood flow from hypovolemia as opposed to direct nephrotoxicity, and they are often treated with IV fluids to restore flow [5, 6]. Patients experiencing severe AKI may progress to acute tubular necrosis (ATN) and acute renal failure [5, 6]. Our patient may have experienced ATN and progressed to ARF due to direct nephrotoxicity of tobramycin and vancomycin as they reached nephrotoxic levels within the serum [5, 6, 10, 14, 22]. Treatment for ARF includes determining the etiology and treating or removing inciting factors, optimizing fluids and electrolytes and occasionally hemodialysis [5, 6]. In case reports and small series, patients experiencing ARF due to antibiotics spacer toxicity required multiple sessions of dialysis and eventually antibiotic spacer explantation before kidney function was restored [5]. We dialyzed our patient initially for hyperkalemia with ECG changes but repeated it for elimination of tobramycin and vancomycin. Upon the cessation of hemodialysis, tobramycin and creatinine began to reaccumulate in the serum suggesting conservative management would not be adequate. Subsequently the patient underwent surgical explantation of the spacer.

Overall, our patient experienced significant morbidity secondary to his AKI. Many factors may be implicated in this outcome, but it is unclear which are significant. Based on the Naranjo Score (=6), our patient most likely experienced AKI secondary to the vancomycin and tobramycin antibiotic spacer. More studies are needed to further delineate elution profiles of spacers as well as perioperative and patient risk factors contributing to AKI during revision arthroplasty.

## 4. Conclusion

Uses of tobramycin and vancomycin antibiotic spacers used in two-stage revision arthroplasty have potentially severe complications of acute renal failure. Physicians and pharmacists should have a high level of suspicion of spacers causing AKI and be aware of the risk of ARF during the postoperative period in patients undergoing revision arthroplasty.
